# Continuous-Variable Quantum Fourier Neural Operator for Solving Partial Differential Equations

**DOI:** 10.3390/e28070737

**Published:** 2026-07-01

**Authors:** Paolo Marcandelli, Stefano Mariani, Martina Siena, Stefano Markidis

**Affiliations:** 1 Department of Civil and Environmental Engineering, Politecnico di Milano, 20133 Milan, Italy; stefano.mariani@polimi.it (S.M.); martina.siena@polimi.it (M.S.); 2 Department of Computational Science and Technology, KTH Royal Institute of Technology, 114 28 Stockholm, Sweden

**Keywords:** Fourier neural operator, Continuous-Variable Quantum Computing, Photonic machine learning, neural operator, partial differential equations

## Abstract

Fourier Neural Operators have become a central tool for learning solution operators of partial differential equations, but their spectral layers remain entirely classical and rely on digital Fourier processing. In this work, we introduce the Continuous-Variable Quantum Fourier Neural Operator (CV-QFNO), a Gaussian photonic formulation of the FNO spectral layer. The proposed architecture maps the essential operations of Fourier-domain operator learning, Fourier transformation, mode selection, and channel mixing, onto native continuous-variable optical primitives. In this way, the CV-QFNO provides a photonic quantum analogue of the truncated spectral mechanism underlying the classical FNO, while avoiding the compilation overhead and spectral mismatch that arise in qubit-based Quantum FNO constructions. We extended the framework to both one- and two-dimensional operator learning and validated it on standard PDE benchmarks, including Burgers’ equation, heat equation, Navier–Stokes dynamics, and Darcy flow. The results show that the proposed model preserves the predictive accuracy, resolution generalisation, and spectral inductive bias of Fourier neural operators while using structurally constrained photonic parameterisation. Since all the experiments were performed as classical simulations, the contribution should be understood as an architectural and algorithmic blueprint for photonic neural operators rather than as a demonstration of quantum computational advantage.

## 1. Introduction

Partial differential equations (PDEs) are central to the mathematical modelling of physical and engineering systems, but their numerical solution can become computationally demanding in regimes requiring repeated simulations, real-time prediction, uncertainty quantification, or high-dimensional parametric studies. This has motivated the development of learning-based surrogate models, and in particular neural operators, which aim to approximate solution operators between function spaces rather than individual solution instances [1,2,3]. Among them, the Fourier Neural Operator (FNO) has become a particularly influential architecture for PDE learning, since it parameterises the nonlocal part of the operator in Fourier space, where many PDE dynamics admit a compact spectral representation [2].

The spectral structure of the FNO makes it especially relevant from the perspective of quantum computing. Each FNO layer applies a Fourier transform, performs a learnable transformation on a finite set of retained modes, suppresses the remaining modes, and maps the result back to physical space through the inverse Fourier transform. Thus, the FNO spectral layer is not merely a neural network block but a truncated spectral operator. This structure suggests a natural route toward quantum and photonic implementations, where Fourier transforms and mode-wise operations may be embedded directly into the computational substrate rather than treated as external classical routines.

Qubit-based Quantum Fourier Neural Operators have recently explored this direction by combining unary encodings, Hamming-weight-preserving circuits, and quantum Fourier transforms [4,5,6]. In this setting, data are represented in fixed-Hamming-weight subspaces, and trainable spectral mixing layers are realised through orthogonal transformations implemented by Reconfigurable Beam Splitter (RBS) gates. This formulation has three important advantages. First, the unary-QFT provides a structured quantum implementation of the Fourier transform: while its gate count scales as $O(N_{s}\log N_{s})$, analogously to the classical FFT, its circuit depth can scale as $O(\log N_{s})$ under suitable hardware assumptions allowing parallel operations on disjoint qubit pairs. Second, the architecture provides a direct quantum analogue of the Fourier layer by combining Fourier-domain processing with mode-wise trainable transformations. Third, it constrains the trainable spectral weights to orthogonal matrices, reducing the number of parameters with respect to the unconstrained complex tensors used in classical FNOs. Orthogonal parameterisations are also known to improve conditioning, gradient propagation, and training stability in classical and quantum learning models [7,8,9].

However, the qubit formulation also presents two structural limitations. First, RBS gates are not native operations on standard qubit hardware and must be compiled into elementary one- and two-qubit gates, introducing a substantial entangling-gate overhead. This makes full QFNO circuits difficult to realise on near-term qubit devices. Second, the parallel QFNO construction does not exactly reproduce the truncated spectral action of the classical FNO: while the selected Fourier modes are processed by trainable circuits, the non-selected modes remain present in the quantum state instead of being set to zero [5]. This introduces a systematic spectral mismatch with the classical FNO layer, whose high-frequency modes are explicitly filtered before applying the inverse Fourier transform.

Continuous-variable (CV) quantum computing provides a natural framework to address both issues. In photonic CV systems, information is carried by bosonic modes and processed through Gaussian operations such as beam splitters, phase shifters, squeezers, and loss channels [10,11,12,13]. These operations are directly suited to spectral processing: the Cooley–Tukey structure of the Fourier transform can be mapped onto optical networks of beam splitters and phase rotations, while trainable orthogonal mixing can be implemented by passive interferometers. Moreover, unwanted Fourier modes can be physically suppressed by routing them through loss channels with transmissivity η=0, thereby recovering the explicit truncation mechanism of the classical FNO.

In this work, we introduce the Continuous-Variable Quantum Fourier Neural Operator (CV-QFNO), a Gaussian photonic formulation of the FNO spectral layer. The construction builds on the continuous-variable quantum Fourier layer introduced in [14], where a full matrix is embedded into the off-diagonal covariance block of a bipartite Gaussian state and Fourier transformations are implemented through optical Gaussian circuits. Here, we extend that framework from a fixed Fourier processing layer to a trainable neural-operator architecture. In the proposed CV-QFNO, Fourier transformation, mode selection, and channel mixing are all mapped onto native CV optical primitives: optical quantum Fourier transforms perform the spectral change of basis, zero-transmissivity loss channels remove non-retained modes, and pyramidal interferometers implement trainable orthogonal mixing across channels.

This formulation should not be interpreted as a claim of quantum computational advantage. All the experiments in this work were performed as classical simulations of the corresponding Gaussian transformations. Rather, the contribution is architectural and algorithmic: we show that continuous-variable photonic formalism provides a structurally faithful implementation of the FNO spectral mechanism, while avoiding the qubit compilation overhead and the spectral mismatch of previous QFNO constructions.

The main contributions of this work are as follows:

- We introduce the CV-QFNO, a Gaussian photonic formulation of the Fourier Neural Operator spectral layer in which Fourier transformation, mode selection, and channel mixing are expressed in terms of native continuous-variable optical operations.
- We show that the parallel qubit QFNO suffers from a systematic spectral mismatch with the classical FNO, since non-selected Fourier modes are left unchanged. The CV-QFNO removes this mismatch by using loss channels with transmissivity η=0, thereby recovering the truncated spectral structure of the classical FNO.
- We develop both one- and two-dimensional CV-QFNO architectures. The one-dimensional construction assigns each retained Fourier mode to an independent optical branch, while the two-dimensional extension introduces cross-circuit interferometric coupling to realise channel mixing across spatial Fourier modes.
- We provide a resource comparison between qubit and continuous-variable formulations, highlighting that the qubit implementation requires a large number of compiled entangling gates, whereas the CV construction is naturally expressed through passive linear-optical elements.
- We benchmark the FNO, QFNO, and CV-QFNO on representative PDE operator-learning tasks, including Burgers’ equation, heat equation, Navier–Stokes dynamics, and Darcy flow. The results show that the CV-QFNO preserves the accuracy and resolution-generalisation behaviour of the FNO while using a constrained orthogonal spectral parameterisation.

## 2. Methods

### 2.1. Classical Fourier Neural Operator

We briefly recall the classical Fourier Neural Operator (FNO) [2], focusing only on the spectral structure that will serve as the reference target for the qubit and continuous-variable formulations developed below. In the neural-operator framework, the goal is to approximate a solution operator

$$G^{†}:A\to U,$$

mapping input functions to output functions. Given an input $a:D_{j}\to R^{d_{a}}$, the FNO first lifts it pointwise to a latent field $v_{t}:D_{j}\to R^{d_{v}}$, where *t* indexes the Fourier layer within the stacked FNO architecture, i.e., $v_{t}$ denotes the latent field before the *t*-th Fourier block and $v_{t+1}$ the corresponding output. The latent representation is then updated by Fourier layers of the form

(1) $$v_{t+1}\left(x\right)=\sigma \;\left(Wv_{t}\left(x\right)+F^{-1}\;\left(R_{\phi }\cdot F\left(v_{t}\right)\right)\left(x\right)\right),$$

where *W* is a learnable pointwise linear map, σ is a nonlinear activation function, and F, $F^{-1}$ denote the Fourier and inverse Fourier transforms along the spatial variables. The symbol “·” denotes the mode-wise action of the spectral weights on the Fourier coefficients; in discrete form this corresponds to a matrix–vector or tensor contraction over the channel dimension.

We now make Equation (1) explicit in one and two spatial dimensions. This is not intended as a full review of FNOs, but as a preparation for the quantum constructions in Section 2.2 and Section 2.3: both architectures operate on matrix or tensor representations of the latent field, and must reproduce the Fourier transform, the mode-wise channel mixing, and the suppression of non-retained modes.

#### 2.1.1. One-Dimensional Case

We first consider the common one-dimensional structure underlying the Burgers’ and heat-equation benchmarks reported in Section 3. Their specific PDE definitions and numerical settings are given there; here we only need the latent representation on which the FNO spectral layer acts. For both 1D benchmarks, the lifting map *P* promotes the input $a:D_{j}\to R^{d_{a}}$ to the latent field $v_{t}:D_{j}\to R^{d_{v}}$, evaluated at each of the $N_{s}$ grid points ${\left\{x_{j}\right\}}_{j=1}^{N_{s}}$. Collecting these evaluations column-wise defines the latent matrix

(2) $$A=\left(\begin{array}{cccc} v_{t}\left(x_{1}\right) & v_{t}\left(x_{2}\right) & \cdots & v_{t}\left(x_{N_{s}}\right) \end{array}\right)\in R^{N_{c}\times N_{s}},\;N_{c}=d_{v},$$

where each column $v_{t}\left(x_{j}\right)\in R^{N_{c}}$ is the channel vector at spatial location $x_{j}$. Applying the Fourier transform F along the spatial direction transforms each row of *A*, producing

(3) $$\hat{A}=\left(\begin{array}{cccc} {\hat{v}}_{t}\left(1\right) & {\hat{v}}_{t}\left(2\right) & \cdots & {\hat{v}}_{t}\left(N_{s}\right) \end{array}\right)\in C^{N_{c}\times N_{s}},$$

where the hat denotes quantities represented in Fourier space. Thus, $\hat{A}$ is the Fourier-space representation of the latent matrix *A*, obtained by applying F along the spatial direction, and ${\hat{v}}_{t}\left(k\right)\in C^{N_{c}}$ collects the *k*-th Fourier coefficient across all channels simultaneously. The learnable operator $R_{\phi }^{\left(k\right)}\in C^{N_{c}\times N_{c}}$ then acts along the channel direction on each retained mode independently,

(4) $${\hat{v}}_{t}\left(k\right)\;\mapsto \;R_{\phi }^{\left(k\right)}\;{\hat{v}}_{t}\left(k\right),\;k=1,\ldots ,K,$$

while all non-retained modes are set to zero. Since $R_{\phi }^{\left(k\right)}$ is an unconstrained matrix in $C^{N_{c}\times N_{c}}$, writing Rϕ(k)=Re(Rϕ(k))+iIm(Rϕ(k)), the product $R_{\phi }^{\left(k\right)}{\hat{v}}_{t}\left(k\right)$ *cross-mixes* the real and imaginary parts of the mode vector:

(5) ReRϕ(k)v^t(k)=Re(Rϕ(k))Re(v^t(k))−Im(Rϕ(k))Im(v^t(k)),ImRϕ(k)v^t(k)=Im(Rϕ(k))Re(v^t(k))+Re(Rϕ(k))Im(v^t(k)).

The two output components are therefore independent linear combinations of *both* input components, with no structural coupling between Re(Rϕ(k)) and Im(Rϕ(k)). This unconstrained complex parameterisation uses $2N_{c}^{2}$ real degrees of freedom per retained mode. The resulting filtered Fourier matrix is

(6) $${\hat{A}}^{\prime }=\left(\begin{array}{cccccc} R_{\phi }^{\left(1\right)}{\hat{v}}_{t}\left(1\right) & \cdots & R_{\phi }^{\left(K\right)}{\hat{v}}_{t}\left(K\right) & 0 & \cdots & 0 \end{array}\right)\in C^{N_{c}\times N_{s}},$$

where the last $N_{s}-K$ columns are zeroed out. Since $F^{-1}$ acts along the spatial direction, i.e., row-wise across the $N_{s}$ columns of ${\hat{A}}^{\prime }$, it can be applied outside the matrix to recover the output latent matrix,

(7) $$A^{\prime }=F^{-1}\;\left({\hat{A}}^{\prime }\right)\in R^{N_{c}\times N_{s}},\;A^{\prime }=\left(\begin{array}{ccc} v_{t+1}\left(x_{1}\right) & \cdots & v_{t+1}\left(x_{N_{s}}\right) \end{array}\right),$$

whose *j*-th column $v_{t+1}\left(x_{j}\right)$ is the updated channel vector at spatial location $x_{j}$, consistent with Equation (1) evaluated at $x\in D_{j}\subset R$.

#### 2.1.2. Two-Dimensional Case

For two-dimensional operator-learning problems, such as the Navier–Stokes and Darcy-flow benchmarks reported in Section 3, the latent field is evaluated on an $N_{x}\times N_{y}$ spatial grid. After lifting, we collect the channel values at each spatial location into a tensor $A\in R^{N_{c}\times N_{x}\times N_{y}}$, with $N_{c}=d_{v}$, defined by

(8) $$A_{:,i,j}=v_{t}\left(x_{i},y_{j}\right)\in R^{N_{c}},\;i=1,\ldots ,N_{x},\;j=1,\ldots ,N_{y}.$$

Here the colon in $A_{:,i,j}$ denotes all entries along the channel dimension: for each spatial grid point $\left(x_{i},y_{j}\right)$, the slice $A_{:,i,j}$ is the $N_{c}$-dimensional vector collecting all latent channels at that location. Applying the 2D Fourier transform $F_{2D}$ along both spatial directions transforms each channel independently, producing

(9) $${\hat{A}}_{:,k_{1},k_{2}}={\hat{v}}_{t}\left(k_{1},k_{2}\right)\in C^{N_{c}},\;k_{1}=1,\ldots ,N_{x},\;k_{2}=1,\ldots ,N_{y},$$

where ${\hat{v}}_{t}\left(k_{1},k_{2}\right)$ collects the $\left(k_{1},k_{2}\right)$-th 2D Fourier coefficients across all channels simultaneously. The learnable operator $R_{\phi }^{\left(k_{1},k_{2}\right)}\in C^{N_{c}\times N_{c}}$ then acts along the channel direction on each retained mode pair independently,

(10) $${\hat{v}}_{t}\left(k_{1},k_{2}\right)\;\mapsto \;R_{\phi }^{\left(k_{1},k_{2}\right)}\;{\hat{v}}_{t}\left(k_{1},k_{2}\right),\;k_{1}=1,\ldots ,K_{x},\;k_{2}=1,\ldots ,K_{y},$$

while all non-retained mode pairs are set to zero. As in the 1D case (cf. Equation (5)), the unconstrained complex matrix $R_{\phi }^{\left(k_{1},k_{2}\right)}\in C^{N_{c}\times N_{c}}$ cross-mixes the real and imaginary parts of each retained 2D Fourier mode vector, with $2N_{c}^{2}$ real degrees of freedom per retained mode pair. The resulting filtered Fourier tensor is

(11) $${\hat{A}}_{:,k_{1},k_{2}}^{\prime }=\left\{\begin{array}{cc} R_{\phi }^{\left(k_{1},k_{2}\right)}\;{\hat{v}}_{t}\left(k_{1},k_{2}\right) & k_{1}\leq K_{x},\;k_{2}\leq K_{y},\\ 0 & \mathrm{otherwise}, \end{array}\right.$$

with ${\hat{A}}^{\prime }\in C^{N_{c}\times N_{x}\times N_{y}}$. Since $F_{2D}^{-1}$ acts along both spatial directions, it can be applied outside the tensor to recover the output latent field,

(12) $$\begin{array}{cc} A^{\prime } & =F_{2D}^{-1}\;\left({\hat{A}}^{\prime }\right)\in R^{N_{c}\times N_{x}\times N_{y}},\\ A_{:,i,j}^{\prime } & =v_{t+1}\left(x_{i},y_{j}\right), \end{array}$$

whose (i,j)-th slice $v_{t+1}\left(x_{i},y_{j}\right)$ is the updated channel vector at spatial location $\left(x_{i},y_{j}\right)$, consistent with Equation (1) evaluated at $\left(x,y\right)\in D_{j}\subset R^{2}$.

### 2.2. Quantum FNO: Qubit Formulation

In this section we review the qubit-based Quantum Fourier Neural Operator introduced in [5], which provides the direct precursor to our continuous-variable formulation. We present the core theoretical ingredients of the architecture, namely data encoding, the unary quantum Fourier transform, and the learnable spectral mixing layer, and we then examine the two structural limitations that motivate the transition to a continuous-variable framework: the hardware compilation overhead inherent to the qubit setting, and the systematic spectral mismatch with the classical FNO introduced by the parallel circuit variant. The notation for the latent representation depends on the spatial dimensionality of the problem: in one dimension the latent field is collected into a matrix $A\in R^{N_{c}\times N_{s}}$, whereas in two dimensions it is collected into a tensor $A\in R^{N_{c}\times N_{x}\times N_{y}}$. The following treatment, as presented in [5], focuses on the one-dimensional setting, so that the latent representation is the matrix *A* defined in Equation (2). For an extension to higher-dimensional domains we refer the reader to [6].

#### 2.2.1. Data Encoding

The goal of the encoding step is to embed the latent matrix $A\in R^{N_{c}\times N_{s}}$ defined in Equation (2) into a quantum state, so that subsequent quantum operations reproduce the spectral mixing of Equation (4) at the quantum level. This matrix is encoded as a quantum state in the *unary basis* by constructing the two-register state

(13) $$|A\rangle =\frac{1}{∥A∥}\sum_{i=1}^{N_{c}}\sum_{j=1}^{N_{s}}a_{ij}\;|e_{j}\rangle |e_{i}\rangle ,$$

where $|e_{k}\rangle$ denotes the *k*-th unary basis vector (a computational basis state with a single 1 at position *k*). This encoding requires $N_{c}+N_{s}$ qubits and can be loaded with a butterfly circuit with depth $O(\log N_{c}+2N_{c}\log N_{s})$ and gate count $\left(N_{c}-1\right)+\left(2N_{c}-1\right)\left(N_{s}-1\right)$. See [5,15] for more information about unary encoding and circuit depth.

#### 2.2.2. Unary Quantum Fourier Transform

The classical FFT butterfly structure is reproduced quantumly via a circuit composed of single-qubit phase gates and parameterised two-qubit Reconfigurable Beam Splitter (RBS) gates [5]. Applied to the lower ($N_{s}$-qubit) register of the encoded state |A〉 in Equation (13), this *Unary-QFT* implements the row-wise discrete Fourier transform of *A* entirely within the unary subspace. The full two-register action is

(14) $$\mathrm{QFT}\;|A\rangle =\frac{1}{∥A∥}\sum_{i=1}^{N_{c}}\mathrm{QFT}\;\left(\sum_{j=1}^{N_{s}}a_{ij}\;|e_{j}\rangle \right)|e_{i}\rangle =\frac{1}{∥A∥}\sum_{i=1}^{N_{c}}\sum_{j=1}^{N_{s}}{\hat{a}}_{ij}\;|e_{j}\rangle |e_{i}\rangle =:|\hat{A}\rangle ,$$

where ${\hat{a}}_{ij}$ are the Fourier coefficients of the *i*-th row of *A*, so that the quantum state $|\hat{A}\rangle$ encodes the transformed matrix defined in Equation (3). This is the quantum analogue of applying F row-wise to *A* along the spatial direction: the channel index *i* (upper register) is untouched, while the spatial index *j* (lower register) is transformed, in direct correspondence with the classical operation $A\mapsto \hat{A}$. The circuit requires $O(N_{s}\log N_{s})$ gates and runs in depth $O(\log N_{s})$ when disjoint qubit pairs can be addressed in parallel, as in trapped-ion or cold-atom hardware.

#### 2.2.3. Parallel Quantum Fourier Layer and Its Limitations

**Figure 1:**
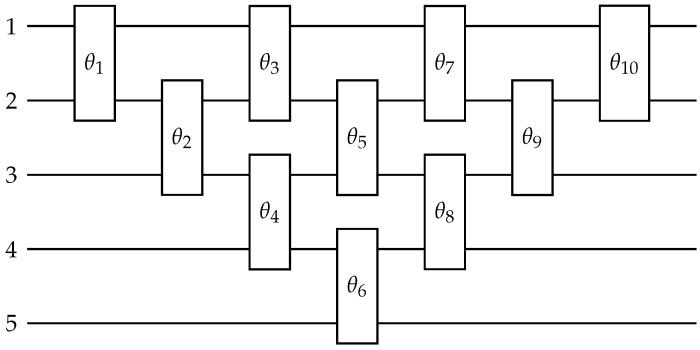
Pyramidal interferometer on $N_{c}=5$ modes implementing an arbitrary 5 × 5 orthogonal matrix in the unary subspace. Each box represents an $\mathrm{RBS}(\theta_{k})$ gate with an independent trainable parameter $\theta_{k}$. Gates in the same column act on disjoint wire pairs and run in parallel. The circuit uses $\frac{N_{c}\left(N_{c}-1\right)}{2}=10$ gates arranged in $2N_{c}-3=7$ sequential timesteps, with at most $\lfloor N_{c}/2\rfloor =2$ gates per timestep, achieving circuit depth $O(N_{c})$.

The architecture we describe is the Parallel QFL of [5]. After the Unary-QFT has mapped the encoded state |A〉 to $|\hat{A}\rangle$ (see Equation (14)), the intended operation is to reproduce the filtered spectral matrix of Equation (6). This is done, at the level of the target FNO layer, by applying an independent learnable mixing $R_{\phi }^{\left(k\right)}$ to each of the *K* retained Fourier modes and setting the remaining $N_{s}-K$ modes to zero. To this end, *K* independent quantum circuits are prepared, one for each retained mode k∈{1,…,K}. In circuit *k*, a pyramidal interferometer $W_{Q}^{k}$ (Figure 1) is applied to the upper ($N_{c}$-qubit) register, controlled on the *k*-th qubit of the lower register: the gate fires only when the lower register is in state $|e_{k}\rangle$, i.e., only for Fourier mode *k*.

Since each RBS(θ) gate restricted to the unary subspace acts as a Givens rotation $G_{ij}\left(\theta \right)\in O\left(N_{c}\right)$ and any orthogonal matrix admits a decomposition into at most $\frac{N_{c}\left(N_{c}-1\right)}{2}$ such rotations [16], the pyramidal circuit with one RBS per pair (i,j) provides a universal parameterisation of $O(N_{c})$ [17]. The resulting weight matrices ${\left\{W_{Q}^{k}\right\}}_{k=1}^{K}$ are therefore fully learnable orthogonal operators. This is a deliberate architectural choice: by restricting the spectral mixing to $O\left(N_{c}\right)\subset U\left(N_{c}\right)$ the same real matrix $W_{Q}^{k}$ is applied independently to the real and imaginary parts of each Fourier mode vector

(15) Re(WQkv^t(k))=WQkRe(v^t(k)),Im(WQkv^t(k))=WQkIm(v^t(k)),

without the cross-coupling between real and imaginary components that characterises the classical FNO complex weight (cf. Equation (5)). The orthogonality constraint reduces the parameter count per mode from $2N_{c}^{2}$ to $\frac{N_{c}\left(N_{c}-1\right)}{2}$, while improving gradient conditioning and reducing overfitting [7,8,9].

While the restriction to $O(N_{c})$ can in principle be relaxed to $U(N_{c})$ to recover the cross-mixing structure of the classical FNO (cf. Equation (5)), we retain the real orthogonal parameterisation for the training-efficiency reasons discussed in Section 2.4. The effective action of circuit *k* is

(16) $$|\hat{A}\rangle \;⟼\;\frac{1}{∥A∥}\sum_{i=1}^{N_{c}}\sum_{j=1}^{N_{s}}{\tilde{a}}_{ij}^{\left(k\right)}\;|e_{j}\rangle |e_{i}\rangle ,\;{\tilde{a}}_{ij}^{\left(k\right)}=\left\{\begin{array}{cc} {\left(W_{Q}^{k}\;{\hat{v}}_{t}\left(k\right)\right)}_{i} & j=k,\\ {\left({\hat{v}}_{t}\left(j\right)\right)}_{i} & j\neq k. \end{array}\right.$$

After applying the Unary-IQFT to the lower register, the output of circuit *k* is

(17) $${\mathrm{output}}_{k}=F^{-1}\;\left[{\hat{v}}_{t}\left(1\right),\ldots ,W_{Q}^{k}{\hat{v}}_{t}\left(k\right),\ldots ,{\hat{v}}_{t}\left(N_{s}\right)\right],$$

which modifies only mode *k* but carries the full Fourier content of all the other modes j≠k. Summing the *K* circuit outputs therefore does not reproduce the classical FNO target $A^{*}=F^{-1}\left[W_{Q}^{1}{\hat{v}}_{t}\left(1\right),\ldots ,W_{Q}^{K}{\hat{v}}_{t}\left(K\right),0,\ldots ,0\right]$: each circuit contributes a spurious background from the non-selected modes, and the sum accumulates *K* such copies. To see this explicitly, decompose each output by linearity of $F^{-1}$:

(18) $$\begin{array}{cc} {\mathrm{output}}_{k} & =F^{-1}\;\left[{\hat{v}}_{t}\left(1\right),\ldots ,{\hat{v}}_{t}\left(N_{s}\right)\right]+F^{-1}\;\left[0,\ldots ,\left(W_{Q}^{k}-I\right){\hat{v}}_{t}\left(k\right),\ldots ,0\right]\\ \; & =A+F^{-1}\;\left[\Delta {\hat{A}}^{\left(k\right)}\right], \end{array}$$

where $\Delta {\hat{A}}^{\left(k\right)}\in C^{N_{c}\times N_{s}}$ has $\left(W_{Q}^{k}-I\right){\hat{v}}_{t}\left(k\right)$ in column *k* and zero elsewhere. Sum over all *K* circuits and apply linearity of $F^{-1}$ to collect the $\Delta {\hat{A}}^{\left(k\right)}$ terms:

(19) $$\begin{array}{cc} \sum_{k=1}^{K}{\mathrm{output}}_{k} & =K\cdot A+F^{-1}\;\left[\sum_{k=1}^{K}\Delta {\hat{A}}^{\left(k\right)}\right]\\ \; & =K\cdot A+F^{-1}\;\left[W_{Q}^{1}{\hat{v}}_{t}\left(1\right),\ldots ,W_{Q}^{K}{\hat{v}}_{t}\left(K\right),0,\ldots ,0\right]-F^{-1}\;\left[{\hat{v}}_{t}\left(1\right),\ldots ,{\hat{v}}_{t}\left(K\right),0,\ldots ,0\right]\\ \; & =A^{*}+\left(K-1\right)\cdot A+F^{-1}\;\left[0,\ldots ,0,\;{\hat{v}}_{t}\left(K+1\right),\ldots ,{\hat{v}}_{t}\left(N_{s}\right)\right], \end{array}$$

where $A^{*}=F^{-1}\left[W_{Q}^{1}{\hat{v}}_{t}\left(1\right),\ldots ,W_{Q}^{K}{\hat{v}}_{t}\left(K\right),0,\ldots ,0\right]$ is the classical FNO target defined in Equation (7), and we use $A=A_{K}+F^{-1}\left[0,\ldots ,0,{\hat{v}}_{t}\left(K+1\right),\ldots ,{\hat{v}}_{t}\left(N_{s}\right)\right]$ with $A_{K}=F^{-1}\left[{\hat{v}}_{t}\left(1\right),\ldots ,{\hat{v}}_{t}\left(K\right),0,\ldots ,0\right]$. The deviation from $A^{*}$ has two distinct sources: (K−1)·A, arising from the K−1 spurious background copies accumulated across circuits, and the residual contribution of the non-selected modes k>K, which are never zeroed.

Note that if in each circuit *k* all modes j≠k were set to zero before the IQFT, the output of circuit *k* would reduce to $F^{-1}\left[0,\ldots ,W_{Q}^{k}{\hat{v}}_{t}\left(k\right),\ldots ,0\right]$ and the sum over *K* circuits would give exactly $A^{*}$ by linearity of $F^{-1}$. This is precisely the fix introduced in Section 2.3: routing the non-selected modes through loss channels with transmissivity η=0 erases their contribution before summation, restoring exact equivalence with Equations (7) and (12).

In terms of circuit resources, the *K* pyramidal interferometers run on independent registers and therefore contribute a total of $K\cdot \frac{N_{c}\left(N_{c}-1\right)}{2}$ RBS gates, equivalently $K\cdot N_{c}\left(N_{c}-1\right)$ CZ gates, at an overall depth of $2N_{c}-3=O\left(N_{c}\right)$, since all *K* circuits execute in parallel.

#### 2.2.4. RBS Gate and Hardware Overhead

Throughout the whole QFNO implementation, the elementary two-qubit operation is the RBS gate. This gate is not native to any current qubit platform and must be compiled into hardware primitives, incurring a substantial overhead that places the full QFNO beyond near-term reach and motivates the continuous-variable reformulation of Section 2.3. The RBS gate at angle θ acts on two qubits as

(20) $$\mathrm{RBS}\left(\theta \right)=\left(\begin{array}{cccc} 1 & 0 & 0 & 0\\ 0 & \cos\theta & \sin\theta & 0\\ 0 & -\sin\theta & \cos\theta & 0\\ 0 & 0 & 0 & 1 \end{array}\right),$$

which, restricted to the unary subspace {|01〉,|10〉}, implements a 2×2 rotation by θ. On standard qubit hardware there is no native RBS gate, so it must be compiled into elementary operations. A minimal decomposition is

(21) $$\mathrm{RBS}\left(\theta \right)=\left(H\;⊗\;H\right)\;\mathrm{CZ}\;(R_{Y}\left(\theta \right)\;⊗\;R_{Y}\left(-\theta \right))\;\mathrm{CZ}\;\left(H\;⊗\;H\right),$$

requiring 2 entangling (CZ) gates and 6 single-qubit gates per RBS. Here *H* denotes the single-qubit Hadamard gate, which maps computational basis states to equal superpositions, while CZ denotes the controlled-*Z* entangling gate, which applies a phase flip when both qubits are in state |1〉 [18]. Table 1 summarises the depth and CZ-gate count for each phase of a single Parallel QFNO layer.

For the representative choice $N_{c}=N_{s}=32$, K=4 the total number of CZ gates is 8256 per layer at a sequential circuit depth of 396. Current superconducting and trapped-ion processors achieve two-qubit gate fidelities of 99–99.9% [19]. At 99% fidelity the overall circuit fidelity per layer is

$$0.99^{8256}\approx 10^{-36},$$

and even at the optimistic 99.9% level it falls to $0.999^{8256}\approx 2.6\times 10^{-4}$, rendering the output indistinguishable from noise for any practically relevant $\left(N_{c},N_{s}\right)$. This positions the full QFNO squarely beyond near-term NISQ capabilities, motivating the shift to a photonic continuous-variable architecture in which the analogous operations are implemented by passive beam splitters, linear-optics elements that realise exact unitary rotations on the field quadratures without any qubit–qubit entangling interaction. The dominant noise source in photonic platforms is propagation loss [20], which is qualitatively different from and typically far smaller than the coherent error rates of two-qubit gates on current qubit hardware.

In summary, the qubit QFNO has two fundamental limitations: a spectral mismatch with the classical FNO (Equation (19)) and a prohibitive hardware overhead on qubit platforms, despite the RBS gate being natively a linear-optical element. These are the main motivations for the CV-QFNO introduced in Section 2.3.

### 2.3. Continuous-Variable Quantum Fourier Neural Operator

We now introduce the Continuous-Variable Quantum Fourier Neural Operator (CV-QFNO), which constitutes the main architectural contribution of this work. The proposed model reformulates the spectral layer of the Fourier Neural Operator within a continuous-variable photonic framework, using the qubit-based QFNO reviewed in Section 2.2 as its direct conceptual precursor.

The qubit-based QFNO encodes data in the unary subspace and implements the learnable spectral mixing through orthogonal rotations realised by RBS gates. Since an RBS gate is the discrete-variable analogue of a linear-optical beam splitter, the structure of the QFNO already suggests a natural connection with photonic hardware. Moving to a full CV formulation [11] makes this connection explicit: the Fourier transform, the trainable orthogonal mixing, and the suppression of unwanted modes can all be expressed directly in terms of Gaussian photonic operations.

This reformulation is not only a change of computational substrate but also addresses a key limitation of the qubit construction. As shown in Section 2.2, the parallel QFNO architecture leaves the non-selected Fourier modes unchanged, rather than setting them to zero. This produces a systematic mismatch with the classical FNO spectral layer (cf. Equation (19)), whose action is explicitly truncated in Fourier space. In the CV setting, by contrast, unused modes can be erased directly by routing them through loss channels with transmissivity η=0. Physically, this corresponds to sending the corresponding optical modes to a beam dump; mathematically, it removes their correlations with the active register. As a result, the CV-QFNO can reproduce the truncated spectral operator of the classical FNO exactly on the retained modes.

#### 2.3.1. Bipartite Gaussian Encoding and CV-QFT

We recall only the elements of the continuous-variable Fourier layer that are needed to construct the CV-QFNO. The full derivation of the bipartite Gaussian encoding, the TMS-SVD loading procedure, and the Cooley–Tukey optical QFT is given in [14].

The latent matrix $A\in R^{N_{c}\times N_{s}}$ introduced in Equation (2) is encoded into a bipartite Gaussian state with two registers. The first register, denoted by $r_{1}$, contains $N_{c}$ bosonic modes associated with the channel dimension, while the second register, denoted by $r_{2}$, contains $N_{s}$ bosonic modes associated with the spatial dimension. We write $x_{r_{1}},p_{r_{1}}$ for the vectors of position and momentum quadratures of the modes in register $r_{1}$, and $x_{r_{2}},p_{r_{2}}$ for the corresponding quadrature vectors of register $r_{2}$.

Using the TMS-SVD encoding of [14], the singular values of *A* are loaded by independent two-mode squeezing gates, while the left and right singular vectors are implemented by passive interferometers acting on the two registers. The resulting bipartite Gaussian state encodes the latent matrix in the inter-register covariance blocks as

(22) $$\sigma_{x_{r_{1}}x_{r_{2}}}=A,\;\sigma_{p_{r_{1}}p_{r_{2}}}=-A,\;\sigma_{x_{r_{1}}p_{r_{2}}}=\sigma_{p_{r_{1}}x_{r_{2}}}=0.$$

Here $\sigma_{x_{r_{1}}x_{r_{2}}}$ denotes the block of the covariance matrix collecting all second-order correlations between the *x*-quadratures of registers $r_{1}$ and $r_{2}$. Analogously, $\sigma_{p_{r_{1}}p_{r_{2}}}$ collects the inter-register *p*-quadrature correlations, while $\sigma_{x_{r_{1}}p_{r_{2}}}$ and $\sigma_{p_{r_{1}}x_{r_{2}}}$ are the mixed inter-register blocks. Thus, the classical latent matrix *A* is represented exactly as the *x*-quadrature cross-correlation block of the bipartite covariance matrix.

On this representation, Fourier transformations are implemented by applying Cooley–Tukey optical QFT circuits to the corresponding registers. Each butterfly of the classical FFT is mapped to Gaussian photonic operations, namely phase rotations and 50:50 beam splitters, as shown in [14]. Applying the QFT to the spatial register $r_{2}$ implements the Fourier matrix $F_{N_{s}}$ on the $N_{s}$ spatial modes. Since the latent matrix is stored in the cross-covariance block $\sigma_{x_{r_{1}}x_{r_{2}}}=A$, this operation transforms all rows of *A* simultaneously:

(23) $$\sigma_{x_{r_{1}}x_{r_{2}}}^{\prime }=\sigma_{x_{r_{1}}x_{r_{2}}}F_{N_{s}}^{⊤}=AF_{N_{s}}^{⊤}=\hat{A}.$$

This is the one-dimensional Fourier representation used in the 1D CV-QFNO construction, where the Fourier transform acts along the spatial direction while the channel register is left unchanged.

Analogously, applying the QFT to the channel register $r_{1}$ implements $F_{N_{c}}$ on the $N_{c}$ channel modes, giving

(24) $$\sigma_{x_{r_{1}}x_{r_{2}}}^{\prime }=F_{N_{c}}\sigma_{x_{r_{1}}x_{r_{2}}}=F_{N_{c}}A.$$

When QFTs are applied simultaneously to both registers, the two-dimensional Fourier transform is obtained:

(25) $$\hat{A}=F_{N_{c}}AF_{N_{s}}^{⊤}.$$

Since the covariance matrix is real, the complex matrix $\hat{A}$ cannot be stored in a single covariance block. Instead, its real and imaginary parts are distributed across two inter-register blocks,

(26) σxr1xr2′=ReFNcAFNs⊤=Re(A^),σxr1pr2′=ImFNcAFNs⊤=Im(A^).

Equivalently,

(27) $$\hat{A}=\sigma_{x_{r_{1}}x_{r_{2}}}^{\prime }+i\;\sigma_{x_{r_{1}}p_{r_{2}}}^{\prime }.$$

Thus, the CV-QFT provides direct access to the complex Fourier coefficients through covariance blocks of the Gaussian state. For the two-register transform, the optical QFT stage has gate count $O(N_{c}\log N_{c}+N_{s}\log N_{s})$ and depth $O(\log\left(N_{c}N_{s}\right))$, excluding the classical SVD preprocessing and encoding costs discussed in [14].

This covariance-block representation also determines how channel mixing is implemented in the CV-QFNO. A retained Fourier mode *k* is represented by its real and imaginary parts across the two covariance blocks above. Applying a passive interferometer $W^{\left(k\right)}\in O\left(N_{c}\right)$ to the channel register therefore acts on both parts in parallel,

(28) Re(a^(k))↦W(k)Re(a^(k)),Im(a^(k))↦W(k)Im(a^(k)).

Consequently, as in the qubit QFNO of Section 2.2, the CV-QFNO uses real orthogonal spectral mixing matrices rather than unconstrained complex matrices. This reduces the number of trainable parameters from $2N_{c}^{2}$ to $\frac{N_{c}\left(N_{c}-1\right)}{2}$ per retained mode and provides a direct passive-interferometric implementation of the mixing layer. In principle, this restriction could be relaxed to unitary mixing in a more general photonic architecture; here we retain the orthogonal parameterisation for consistency with the qubit formulation and for the training-efficiency reasons discussed in Section 2.4.

The novel contribution of the CV-QFNO lies in faithfully reproducing the action of $R_{\phi }$ in Equation (1): after the Fourier transform, each retained mode *k* must be mixed along the channel dimension by an independent learnable weight matrix, while all non-selected modes are set to zero. The local *W* branch of Equation (1) remains classical and is unchanged; it is the global convolution branch that requires a dedicated optical implementation. We now describe how this is achieved first for a 1D input and then for the 2D case.

#### 2.3.2. 1D Architecture: *K* Parallel Circuits

**Figure 2:**
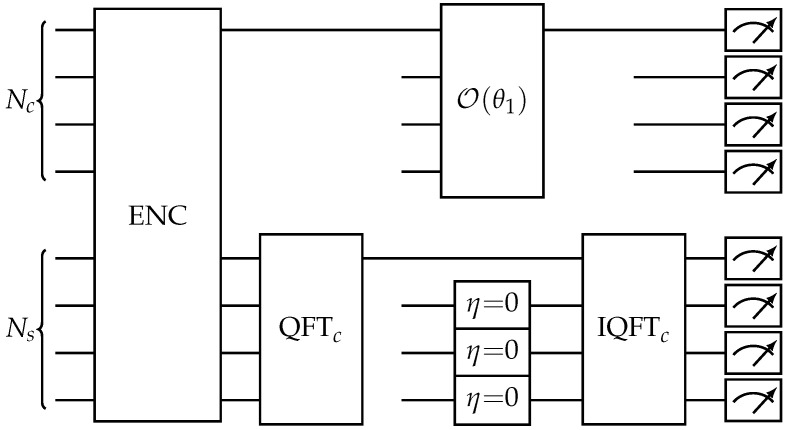
CV-QFNO spectral layer, branch k=1 (1D case). The latent matrix $A\in R^{N_{c}\times N_{s}}$ is encoded via TMS-SVD into the off-diagonal covariance block $\sigma_{x_{r_{1}}x_{r_{2}}}$, with the *n*-mode upper register ($r_{1}$) and the *m*-mode lower register ($r_{2}$). A row-wise ${\mathrm{QFT}}_{c}$ on the lower register places $\hat{A}$ in that block. The pyramidal interferometer $O(\theta_{1})$ is applied to the upper register; loss channels η=0 are applied to all lower-register modes j≠1, leaving mode k=1 active. Each of the remaining K−1 branches is structurally identical, with weight $O(\theta_{k})$ and mode *k* left free. An ${\mathrm{IQFT}}_{c}$ followed by homodyne measurement yields the output contribution of mode k=1.

In a discrete-variable setting, the one- or two-dimensional Quantum Fourier Layer could in principle be implemented through controlled operations on the upper (channel) register conditioned on the lower (spatial) register. To avoid the resulting CNOT-type overhead one may instead adopt the Parallel QFNO strategy of Section 2.3, which directly inspires the present CV construction. However, the CV Gaussian formalism only admits passive linear-optics operations, namely beam splitters and phase shifters, which act jointly on all modes and therefore cannot implement this type of conditional logic. As a result, sequential mode-selective weight application within a single circuit is not available in this framework. To overcome this limitation, we follow the intuition of the Parallel QFNO and run *K* independent optical circuits in parallel, one for each retained Fourier mode *k*. Each circuit receives the same latent matrix *A* and proceeds as follows; a schematic representation is provided in Figure 2.

As shown in Section 2.3, *A* is loaded into a $\left(N_{c}+N_{s}\right)$-mode Gaussian state via TMS-SVD (Equation (22)), setting $\sigma_{x_{r_{1}}x_{r_{2}}}=A$ and $\sigma_{x_{r_{1}}p_{r_{2}}}=0$. Then, ${\mathrm{QFT}}_{c}$ is applied to the lower $N_{s}$-mode register and real and imaginary parts of $\hat{A}$ distribute across two covariance blocks:

(29) σxr1xr2′=Re(A^),σxr1pr2′=Im(A^),

so that $\hat{A}=\sigma_{x_{r_{1}}x_{r_{2}}}^{\prime }+i\;\sigma_{x_{r_{1}}p_{r_{2}}}^{\prime }$, the CV analogue of Equation (3). An orthogonal interferometer $O\left(\theta_{k}\right)\in O\left(N_{c}\right)$ acting on the upper register applies the same real matrix to both blocks simultaneously (cf. Equation (28)), which is the exact photonic analogue of the FNO applying a weight matrix to both the real and imaginary parts of each complex Fourier mode vector. For notational compactness, all subsequent operations in this section are written directly on the full complex matrix $\hat{A}$, with the understanding that each step acts equivalently on both covariance blocks. At this point, the first *K* parallel circuits are assigned independent trainable pyramidal interferometers $O(\theta_{k})$, with one interferometer for each retained Fourier mode *k*.

We now focus on a generic *k*-th circuit; as will be shown below, the full spectral output is then recovered by summing the contributions of all *K* circuits through the linearity of the inverse Fourier transform. In the *k*-th circuit, absent further constraints, applying $O(\theta_{k})$ to the upper $N_{c}$-mode register would mix the channel dimension across all $N_{s}$ Fourier columns simultaneously, yielding

(30) $${\hat{A}}^{\prime (k)}=\left(\begin{array}{ccc} O\left(\theta_{k}\right){\hat{v}}_{t}\left(1\right) & \cdots & O\left(\theta_{k}\right){\hat{v}}_{t}\left(N_{s}\right) \end{array}\right)=O\left(\theta_{k}\right)\hat{A},$$

which does not reproduce the mode-selective operation of Equation (6).

To isolate the *k*-th mode, all lower-register modes j≠k are routed through loss channels with transmissivity η=0, which replace those modes with vacuum and erase their correlations with the upper register. As a result, only the *k*-th column of $\hat{A}$ survives across the active covariance blocks, and the *k*-th parallel circuit produces the filtered Fourier contribution

(31) $${\hat{A}}^{\prime (k)}=\left(\begin{array}{ccccccc} 0 & \cdots & 0 & O\left(\theta_{k}\right){\hat{v}}_{t}\left(k\right) & 0 & \cdots & 0 \end{array}\right)\in C^{N_{c}\times N_{s}},$$

where the only nonzero column is the *k*-th one, thereby realising Equation (4) exactly. The inverse Fourier transform is then applied independently within each parallel circuit. In the *k*-th branch this produces

(32) $$A^{\prime (k)}=F^{-1}\;\left({\hat{A}}^{\prime (k)}\right)\in R^{N_{c}\times N_{s}},$$

and homodyne measurement returns the corresponding contribution to the latent output in the physical domain. The *K* branches are combined only at this stage, by summing their measured outputs:

(33) $$A^{\prime }=\sum_{k=1}^{K}A^{\prime (k)}=\sum_{k=1}^{K}F^{-1}\;\left({\hat{A}}^{\prime (k)}\right).$$

Using the linearity of $F^{-1}$, one obtains

(34) $$A^{\prime }=F^{-1}\;\left(\sum_{k=1}^{K}{\hat{A}}^{\prime (k)}\right)=F^{-1}\;\left(\left(\begin{array}{cccccc} O\left(\theta_{1}\right){\hat{v}}_{t}\left(1\right) & \cdots & O\left(\theta_{K}\right){\hat{v}}_{t}\left(K\right) & 0 & \cdots & 0 \end{array}\right)\right),$$

thereby recovering exactly the filtered spectral structure of Equation (7).

#### 2.3.3. Optical Resource Count

Since the CV-QFNO is built entirely from beam splitters and phase shifters, except for the two-mode squeezing gates used in the encoding step, it incurs no entangling-gate compilation overhead. Table 2 summarises the circuit depth and beam-splitter count associated with each phase of a single CV-QFNO layer.

For the representative choice $N_{c}=N_{s}=32$ and K=4, the above expressions yield a total of 3260 passive beam splitters and 32 two-mode squeezers, at an overall sequential depth of 105. By comparison, the corresponding qubit QFNO layer requires 8256 CZ gates at depth 396 (Table 1). Since beam splitters are low-loss passive optical elements [20], the fidelity argument that renders the qubit implementation impractical does not carry over to the CV setting, making the CV-QFNO a substantially more realistic photonic architecture. However, we note that the TMS-SVD encoding requires a classical singular value decomposition of the input matrix prior to each forward pass, introducing an additional preprocessing cost of order $O(\min{\left(N_{c},N_{s}\right)}^{2}\max\left(N_{c},N_{s}\right))$, which is absent in both the qubit and classical FNO pipelines and should therefore be accounted for in any end-to-end latency comparison.

At the same time, it is important to acknowledge the practical limitations of the present construction. Although the CV-QFNO removes the severe entangling-gate overhead of the qubit formulation, a full hardware realisation of the architecture considered here remains far beyond current photonic quantum technology, especially because the model requires multiple parallel optical branches and precise control of Gaussian operations, loss channels, and readout. In addition, quantum machine learning training remains hybrid, with parameter optimisation performed classically, so that any potential advantage at the circuit level must be weighed against the substantial optimisation overhead of the full training loop. For these reasons, the CV-QFNO should presently be regarded primarily as a theoretical and architectural framework, useful for showing how a Continuous-Variable Quantum Fourier Neural Operator could be constructed in a manner faithful to the spectral structure of the classical FNO. For classical PDE problems on classical hardware, the standard FNO remains by far the most practical and effective choice, and the main value of the CV formulation lies not in immediate applicability, but in clarifying what a coherent photonic quantum analogue of the FNO would look like.

#### 2.3.4. 2D Architecture: $K_{x}K_{y}$ Parallel Branches

In the two-dimensional setting, the latent representation is the tensor $A\in R^{N_{c}\times N_{x}\times N_{y}}$ introduced in Equation (8), where $N_{c}$ denotes the number of latent channels, while $N_{x}$ and $N_{y}$ denote the number of sampled grid points along the two spatial directions. Thus, $A_{l,:,:}\in R^{N_{x}\times N_{y}}$ denotes the two-dimensional spatial field associated with the *l*-th latent channel. Unlike the 1D case, where the latent representation is a matrix with one channel axis and one spatial axis, the 2D tensor has a three-index structure (channel, *x*-spatial, and *y*-spatial). This structure does not admit, within the present Gaussian construction, a direct bipartite encoding that preserves the desired separation between channel mixing and the two spatial Fourier directions.

For this reason, we allocate $N_{c}$ independent optical circuits, one for each channel *l*, and we encode in the *l*-th circuit the spatial slice $A_{l,:,:}\in R^{N_{x}\times N_{y}}$ via TMS-SVD into a bipartite Gaussian state. In this 2D construction, the two registers are denoted by $r_{x}$ and $r_{y}$: $r_{x}$ contains $N_{x}$ bosonic modes associated with the grid points along the *x*-direction, while $r_{y}$ contains $N_{y}$ bosonic modes associated with the grid points along the *y*-direction. By Equation (22), the *x*-quadrature inter-register covariance block in the *l*-th circuit satisfies

(35) $$\sigma_{x_{r_{x}}x_{r_{y}}}^{\left(l\right)}=A_{l,:,:},\;l=1,\ldots ,N_{c}.$$

A two-dimensional CV-QFT is then applied independently within each circuit by acting simultaneously with ${\mathrm{QFT}}_{x}$ on register $r_{x}$ and ${\mathrm{QFT}}_{y}$ on register $r_{y}$, as in Equation (26). Because the 2D DFT result ${\hat{A}}_{l,:,:}=F_{N_{x}}\;A_{l,:,:}\;F_{N_{y}}^{⊤}\in C^{N_{x}\times N_{y}}$ is complex, real and imaginary parts are distributed across two covariance blocks:

(36) σxrxxry(l)⟼Re(A^l,:,:),σxrxpry(l)⟼Im(A^l,:,:),

so that ${\hat{A}}_{l,k_{1},k_{2}}={\left(\sigma_{x_{r_{x}}x_{r_{y}}}^{\left(l\right)}+i\;\sigma_{x_{r_{x}}p_{r_{y}}}^{\left(l\right)}\right)}_{k_{1},k_{2}}$ (cf. Equation (27)). The trainable pyramidal interferometer $O\left(\theta_{k_{1},k_{2}}\right)\in O\left(N_{c}\right)$ acting across the $N_{c}$ circuits applies the same real orthogonal matrix simultaneously to both covariance blocks,

(37) Re(v^t(k1,k2))↦O(θk1,k2)Re(v^t(k1,k2)),Im(v^t(k1,k2))↦O(θk1,k2)Im(v^t(k1,k2)),

the two-dimensional analogue of Equation (28). For notational compactness, the remainder of this section writes all operations on the full complex quantities ${\hat{A}}_{l,:,:}$ and ${\hat{v}}_{t}\left(k_{1},k_{2}\right)$, with the understanding that each step acts equivalently on both covariance blocks.

**Figure 3:**
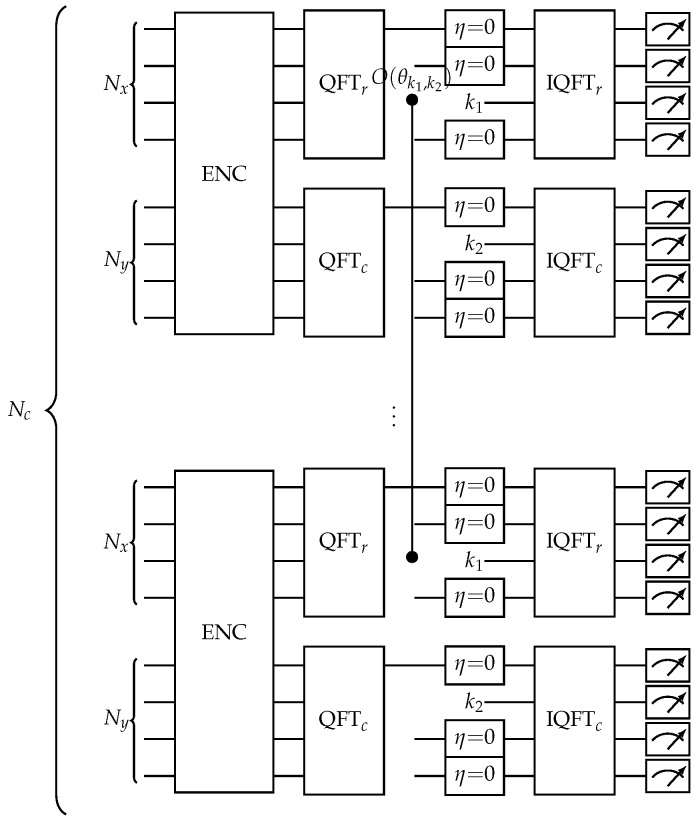
CV-QFNO spectral layer: mode pair $\left(k_{1},k_{2}\right)$ (2D case). $N_{c}$ optical circuits run in parallel, one per channel *l*. Each circuit encodes the 2D slice $A_{l,:,:}$ via TMS-SVD and applies a 2D CV-QFT on both registers. The pyramidal interferometer $O(\theta_{k_{1},k_{2}})$ connects the $k_{1}$-th upper-register wire across all $N_{c}$ circuits, mixing the channel dimension for all column modes. Loss channels η=0 on upper-register modes $i\neq k_{1}$ and lower-register modes $j\neq k_{2}$ then isolate the target mode pair, realising Equation (10). Homodyne measurement yields the output contribution of mode pair $\left(k_{1},k_{2}\right)$.

We now focus on a fixed retained mode pair $\left(k_{1},k_{2}\right)$ with $k_{1}\in \left\{1,\ldots ,K_{x}\right\}$ and $k_{2}\in \left\{1,\ldots ,K_{y}\right\}$; the full output is then recovered by summing the contributions of all the retained mode pairs, as we have previously done in the one-dimensional problem. To isolate the pair $\left(k_{1},k_{2}\right)$, all $r_{x}$-modes $i\neq k_{1}$ and all $r_{y}$-modes $j\neq k_{2}$ are routed through loss channels with transmissivity η=0, see Figure 3. This erases all correlations except those associated with the selected Fourier entry, so that in the *l*-th circuit only the scalar coefficient ${\hat{A}}_{l,k_{1},k_{2}}$ survives. Collecting these coefficients across the $N_{c}$ circuits defines the channel vector

(38) $${\hat{v}}_{t}\left(k_{1},k_{2}\right)={\left({\hat{A}}_{1,k_{1},k_{2}},\ldots ,{\hat{A}}_{N_{c},k_{1},k_{2}}\right)}^{⊤}\in C^{N_{c}}.$$

A trainable pyramidal interferometer $O\left(\theta_{k_{1},k_{2}}\right)\in O\left(N_{c}\right)$ then acts across the $N_{c}$ circuits on the first register ($r_{x}$), connecting the $k_{1}$-th mode wire of each circuit into a shared beam-splitter network. Because $r_{x}$ carries the row index of each covariance block, this action corresponds to a left-multiplication and implements the channel-mixing operation of Equation (10):

(39) $${\hat{v}}_{t}\left(k_{1},k_{2}\right)\;⟼\;O\left(\theta_{k_{1},k_{2}}\right)\;{\hat{v}}_{t}(k_{1},k_{2}).$$

Equivalently, the branch associated with the retained mode pair $\left(k_{1},k_{2}\right)$ produces the filtered Fourier contribution

(40) $${\hat{A}}_{l,i,j}^{\prime (k_{1},k_{2})}=\left\{\begin{array}{cc} {\left[O\left(\theta_{k_{1},k_{2}}\right)\;{\hat{v}}_{t}\left(k_{1},k_{2}\right)\right]}_{l},\; & \left(i,j\right)=(k_{1},k_{2}),\\ 0, & \mathrm{otherwise}, \end{array}\right.$$

that is, a tensor whose only nonzero spatial-frequency entry is the selected mode pair $\left(k_{1},k_{2}\right)$.

The two-dimensional inverse Fourier transform is then applied independently within each branch, yielding the corresponding contribution in the physical domain. After homodyne measurement, the full latent output is recovered by summing the contributions of all retained mode pairs:

(41) $$A^{\prime }=\sum_{k_{1}=1}^{K_{x}}\sum_{k_{2}=1}^{K_{y}}F_{2D}^{-1}\;\left({\hat{A}}^{\prime (k_{1},k_{2})}\right).$$

By linearity of $F_{2D}^{-1}$, this can be written as

(42) $$A^{\prime }=F_{2D}^{-1}\;\left(\sum_{k_{1}=1}^{K_{x}}\sum_{k_{2}=1}^{K_{y}}{\hat{A}}^{\prime (k_{1},k_{2})}\right),$$

which exactly reconstructs the filtered spectral tensor of Equation (12). A schematic representation of a single mode-pair branch is shown in Figure 3.

#### 2.3.5. Optical Resource Count (2D)

The 2D layer comprises $N_{c}\times K_{x}K_{y}$ parallel circuit instances, one per channel per retained mode pair, plus $K_{x}K_{y}$ cross-circuit interferometers for channel mixing. Table 3 summarises the resource budget.

Compared with the 1D case (Table 2), the sequential depth grows only modestly: the 2D QFT replaces a single-register transform with a parallel two-register transform (depth $\max(\log_{2}N_{x},\log_{2}N_{y})$ instead of $\log_{2}N_{s}$), leaving the overall depth dominated by the encoding and channel-mixing stages. However, the beam-splitter count scales as $O(N_{c}K_{x}K_{y}\left(N_{x}^{2}+N_{y}^{2}\right))$, reflecting the $N_{c}$ parallel circuit copies required by the 2D architecture; this additional cost is a direct consequence of the three-index structure of the 2D latent tensor and is shared by any photonic implementation of the 2D operator.

### 2.4. Simulation Setup and Orthogonal Parameterisation

The FNO, QFNO, and the proposed CV-QFNO are algorithmically distinct architectures rooted in different computational substrates and physical principles: classical tensor operations, qubit circuits restricted to a structured Hilbert subspace, and Gaussian photonic modes, respectively. Despite these differences, all three models admit exact classical linear-algebraic representations. The quantum gates and optical transformations appearing in the QFNO and CV-QFNO can therefore be replaced by their corresponding matrix actions, allowing the full architectures to be simulated and trained in PyTorch 2.7.0 with CUDA 12.6 without introducing an additional approximation of the underlying circuit model. This is the standard validation strategy in quantum-inspired machine learning prior to the availability of hardware capable of running the complete architecture at the required scale [4,5,15], and it was the approach adopted in this work.

In the numerical experiments, the three models shared the same overall neural-operator structure, consisting of a lifting map, four spectral blocks, and a final projection. They differed only in the spectral convolution layer, where the quantum-inspired structure enters. In the classical FNO, each retained Fourier mode is mixed across the channel dimension by an unconstrained complex matrix $R_{\phi }^{\left(k\right)}\in C^{N_{c}\times N_{c}}$. In the QFNO and CV-QFNO, this operation is replaced by an orthogonal matrix $W^{\left(k\right)}\in O\left(N_{c}\right)$, reflecting the unitary or passive-interferometric origin of the corresponding quantum transformation. Earlier quantum-inspired networks typically construct such orthogonal matrices as ordered products of $M=N_{c}\left(N_{c}-1\right)/2$ Givens rotations,

$$W=G_{i_{1}j_{1}}\left(\theta_{1}\right)\;G_{i_{2}j_{2}}\left(\theta_{2}\right)\;\cdots \;G_{i_{M}j_{M}}(\theta_{M}),$$

thereby mirroring the gate-by-gate structure of RBS circuits or optical beam-splitter networks [4,15,16]. Although physically natural, this representation is less convenient for classical training, since the matrix *W* must be reconstructed at every forward pass through a sequential product of $O(N_{c}^{2})$ rotation factors.

In this work, both the QFNO and the CV-QFNO instead used a matrix-exponential parameterisation of the orthogonal spectral-mixing matrices. For each retained mode, we introduced a skew-symmetric generator $A\in R^{N_{c}\times N_{c}}$, with $A^{⊤}=-A$, whose independent upper-triangular entries were the trainable parameters:

(43) $$A_{ij}=\theta_{ij},\;A_{ji}=-\theta_{ij}\;\mathrm{for}\;i<j,\;A_{ii}=0,\;W^{\left(k\right)}=\exp(A).$$

This guaranteed orthogonality by construction, since $\exp{\left(A\right)}^{⊤}=\exp\left(A^{⊤}\right)=\exp\left(-A\right)=\exp{\left(A\right)}^{-1}$. The parameterisation used exactly $M=N_{c}\left(N_{c}-1\right)/2$ free parameters, the same number as the Givens decomposition, but avoided the explicit sequential product of rotations during training. In PyTorch 2.7.0, the exponential is evaluated by torch.linalg.matrix_exp, which uses scaling-and-squaring methods with Padé approximants [22], at computational cost $O(N_{c}^{3})$. This cost belongs only to the classical simulation: on a physical CV photonic device, a trained orthogonal transformation would be compiled into beam-splitter and phase-shifter settings, for instance through the Clements decomposition [21], and executed by passive propagation through the interferometric network rather than by explicit matrix exponentiation.

The remaining distinction between the QFNO and the CV-QFNO concerns the treatment of Fourier modes beyond the retained cutoff. In the classical FNO, non-retained modes are set to zero before applying the inverse Fourier transform. The CV-QFNO reproduces this operation exactly in the simulated model by initialising the output spectrum to zero and writing only the retained, orthogonally mixed modes; this is the classical linear-algebraic counterpart of routing the non-selected optical modes through loss channels with transmissivity η=0. The QFNO, by contrast, follows the behaviour of the parallel qubit construction: the full input spectrum is copied, and only the retained modes are overwritten by the mixed values. The modes beyond the cutoff therefore remain unchanged, because they are not explicitly discarded by the qubit circuit. This difference is the source of the systematic spectral contamination discussed in Section 2.2.

The simulations reported here should therefore be interpreted as a classical validation of the proposed architectures, not as a demonstration of quantum computational advantage. Since every quantum or photonic operation is replaced by its matrix equivalent and executed on a GPU, the resulting pipeline is a classical algorithm: it does not realise quantum speedup, exponential state-space compression, or genuine quantum parallelism. Its purpose is architectural. The experiments show that the CV formulation provides a spectrally faithful and hardware-compatible quantum analogue of the FNO layer, with a native mechanism for exact mode suppression and a direct mapping of Fourier and mixing operations onto Gaussian photonic primitives. Any practical computational advantage would require deployment on native photonic hardware, where these transformations are carried out by the physical propagation of light rather than by explicit matrix multiplication, or the use of non-Gaussian resources leading to regimes not efficiently captured by classical Gaussian emulation. These perspectives are discussed further in the conclusion.

All simulations were run on a workstation equipped with an Intel Core i7-14700F processor, 32 GB DDR5 RAM, and an NVIDIA GeForce RTX 4070 GPU with 12 GB of GDDR6X VRAM. PyTorch 2.7.0 training was accelerated on the GPU using CUDA.

## 3. Results

We evaluated the proposed CV-QFNO against two reference architectures: the classical Fourier Neural Operator (FNO) [2] and the qubit-based Quantum Fourier Neural Operator (QFNO) [5]. The comparison was performed on four standard PDE benchmarks: the one-dimensional Burgers’ equation, the one-dimensional heat equation, the two-dimensional Navier–Stokes equation in vorticity form, and the two-dimensional Darcy flow problem. Within each benchmark, all the models are trained using identical data splits, training schedules, and optimisation settings.

The main result of the paper is the formulation of a CV photonic analogue of the FNO spectral layer that faithfully reproduces its truncated Fourier structure. As discussed above, this formulation is particularly natural in CV quantum computing: the Fourier transform, mode filtering, and trainable channel mixing can be implemented directly in terms of Gaussian photonic operations, such as beam splitters, phase shifters, loss channels, and interferometric networks. In this sense, the CV-QFNO provides a hardware-compatible route to a photonic implementation of the FNO spectral mechanism, while avoiding the qubit-gate compilation overhead of discrete-variable QFNO constructions.

Within this perspective, the numerical benchmarks show that CV-QFNO retains competitive predictive accuracy with respect to both FNO and QFNO, while using substantially fewer trainable parameters than the classical model. This parameter reduction follows from the orthogonal parameterisation of the spectral-mixing matrices induced by the pyramidal interferometers. Since CV-QFNO and QFNO use the same orthogonal channel-mixing structure, their trainable-parameter counts were identical in all the experiments.

### 3.1. One-Dimensional Experiments

We first considered two one-dimensional operator-learning benchmarks: the viscous Burgers’ equation,

(44) $$\partial_{t}u\left(x,t\right)+u\left(x,t\right)\;\partial_{x}u\left(x,t\right)=\nu \;\partial_{xx}u\left(x,t\right),\;x\in \left[0,1\right],\;t\in (0,T],$$

and the heat equation,

(45) $$\partial_{t}u\left(x,t\right)=\alpha \;\partial_{xx}u\left(x,t\right),\;x\in \left[0,1\right],\;t\in \left(0,T\right].$$

Both problems were posed on the one-dimensional spatial domain D=[0,1], so d=1, and governed the time evolution of a scalar field u:D×[0,T]→R, where *x* was the spatial coordinate and *t* was time. In Equation (44), u(x,t) denotes the scalar velocity field, ν>0 is the viscosity (set to ν=0.1 in our experiments), while $\partial_{t}u$, $\partial_{x}u$, and $\partial_{xx}u$ denote differentiation with respect to time, the first spatial derivative, and the second spatial derivative, respectively. In Equation (45), u(x,t) represents the temperature field and α>0 is the diffusion coefficient (set to α=0.01 and final time T=1).

In both cases, the learning task was formulated as the approximation of a solution operator mapping an initial condition to the solution at a later time. Following the standard FNO setup, the model input was the initial condition concatenated with the spatial coordinate as a positional feature,

(46) $$a\left(x\right)=\left(u(x,0),\;x\right)\in R^{d_{a}},\;d_{a}=2,$$

and the target output was the scalar solution at the final time,

(47) $$u\left(x,T\right)\in R^{d_{u}},\;d_{u}=1.$$

Thus, each training sample corresponded to an input–output pair

$$a\left(\cdot \right)=\left(u(\cdot ,0),\;\cdot \right)⟼u(\cdot ,T),$$

and the learned operator approximated

$$G^{†}:a\left(\cdot \right)\mapsto u(\cdot ,T).$$

In the implementation, the input was evaluated on a uniform grid of $N_{s}$ spatial points and lifted to a latent representation with $N_{c}$ channels. Therefore, at each Fourier layer, the latent state had the matrix structure $A\in R^{N_{c}\times N_{s}}$ that was introduced in Equation (2).

The Fourier transform was applied along the spatial direction, i.e., across the $N_{s}$ grid points, as in Equation (23), while the spectral mixing acted along the channel dimension on each retained Fourier mode. This was precisely the one-dimensional structure used by the classical FNO, the qubit QFNO, and the CV-QFNO.

The two PDEs probed complementary regimes of operator learning. Burgers’ equation combines nonlinear advection with viscous diffusion and may develop sharp solution features, making it a nonlinear benchmark for spectral operator learning. The heat equation is linear and purely diffusive; in Fourier space, its coefficients decay exponentially in time, making it a controlled benchmark for evaluating whether the learned spectral layer captures dissipative dynamics.

Both one-dimensional experiments used the same training protocol. We used $N_{\mathrm{train}}=200$ input–output pairs and $N_{\mathrm{test}}=20$ test pairs, discretised on a uniform grid of 512 points. All the models were trained for 300 epochs with the Adam optimiser [23], using an initial learning rate of $10^{-3}$, reduced by a factor of two every 100 epochs. For both benchmarks, we analysed the models through three complementary diagnostics: validation loss over training, zero-shot resolution generalisation, and spectral accuracy. The first diagnostic compared the optimisation behaviour of the architectures; the second measured whether a model trained at one resolution could be evaluated on unseen grids; and the third assessed whether the learned operator reproduced the relevant Fourier content of the target dynamics.

**Figure 4:**
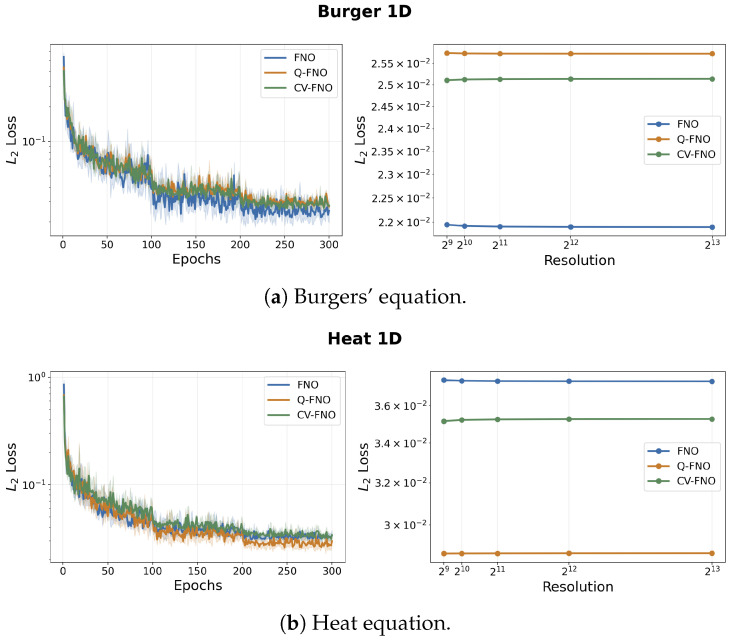
Training dynamics and resolution generalisation: 1D benchmarks. Left: test loss evolution over training epochs. Each solid line shows the geometric mean of the relative $\ell_{2}$ loss across four independent runs (seeds 0, 11, 22, 33); the shaded band spans ±1 geometric standard deviation across the seeds, reflecting run-to-run variability due to random initialisation. Right: relative $\ell_{2}$ test loss as a function of spatial resolution. The models were trained at the base resolution of 512 grid points and evaluated zero-shot at all resolutions shown, without retraining.

Figure 4 reports the loss evolution and resolution generalisation for both one-dimensional benchmarks. To ensure that the quantitative comparisons between architectures reflected genuine structural differences, all the one-dimensional experiments were repeated independently with four different random seeds (0, 11, 22, 33).

For Burgers’ equation, the classical FNO contained 419,009 trainable parameters, whereas QFNO and CV-QFNO each used 122,561, corresponding to a 3.4× reduction. For the heat equation, the FNO used 28,145 parameters, while the QFNO and CV-QFNO used 9329, giving a 3.0× reduction.

This parameter reduction, however, should not be interpreted as a direct wall-clock speedup in the present classical simulations. In the QFNO and CV-QFNO, the quantum or photonic operations are emulated through classical linear algebra, and a substantial part of the cost comes from constructing the orthogonal spectral mixing matrices, as described in Section 2.4. Therefore, training-time comparisons would mix the effect of fewer trainable parameters with the overhead of simulating the quantum-inspired layer. Despite this smaller parameter count, the two quantum-inspired models retained accuracy comparable to the classical FNO, as confirmed by the converged test errors in Table 4a,b. The significance of this result is primarily architectural rather than computational: it shows that the FNO spectral layer can be replaced by a more constrained orthogonal parameterisation without a substantial loss of predictive performance. Such a constraint may improve optimisation stability and become advantageous in future native quantum or photonic implementations.

The loss curves highlight different optimisation behaviours across the two benchmarks. For Burgers’ equation, the FNO converged to a relative $\ell_{2}$ error of $\left(2.52\pm 0.19\right)\times 10^{-2}$ (Table 4a), while the QFNO and CV-QFNO converged to $\left(2.97\pm 0.13\right)\times 10^{-2}$ and $\left(2.93\pm 0.16\right)\times 10^{-2}$, respectively. Despite reaching a slightly lower final test error, the FNO exhibited considerably more unstable training dynamics. Quantifying this over the four independent seeds, the average cross-seed standard deviation of the log test loss, computed epoch-by-epoch across the full training trajectory, was 0.22 for the FNO compared with 0.13 for the QFNO and 0.15 for the CV-QFNO, indicating that the FNO was approximately 70% more sensitive to the random initialisation than the orthogonal models. This proves consistently that the orthogonal spectral-mixing layers produce a substantially smoother and more reproducible optimisation landscape with Burgers’ equation.

For the heat equation, a qualitatively different behaviour emerged. As shown in Figure 4b, the FNO loss curve rose in the final training epochs, a clear sign of overfitting. This is confirmed by the convergence statistics: the FNO reached a test error of $\left(3.27\pm 0.10\right)\times 10^{-2}$ (Table 4b), higher than its own training loss on all four seeds. By contrast, the QFNO converged to the lowest test error of $\left(2.85\pm 0.17\right)\times 10^{-2}$, and the CV-QFNO to $\left(3.39\pm 0.11\right)\times 10^{-2}$, with both orthogonal models showing stable descent throughout training.

Taken together, both one-dimensional benchmarks indicate that constraining the spectral-mixing matrices to the orthogonal group acted as an implicit regulariser: it stabilised the optimisation trajectory across the seeds with Burgers’ equation and prevented overfitting with the heat equation, in both cases without sacrificing predictive accuracy relative to the unconstrained classical FNO.

The resolution-generalisation results, shown in the right panels of Figure 4, confirm that all three architectures preserved the resolution-invariant character expected from neural operators. The models were trained at the base resolution of 512 grid points and evaluated zero-shot at the other resolutions shown, without retraining. Across both the Burgers’ and the heat benchmarks, the QFNO and the CV-QFNO remained competitive with the FNO over the full tested range, with no systematic degradation at unseen spatial resolutions.

**Figure 5:**
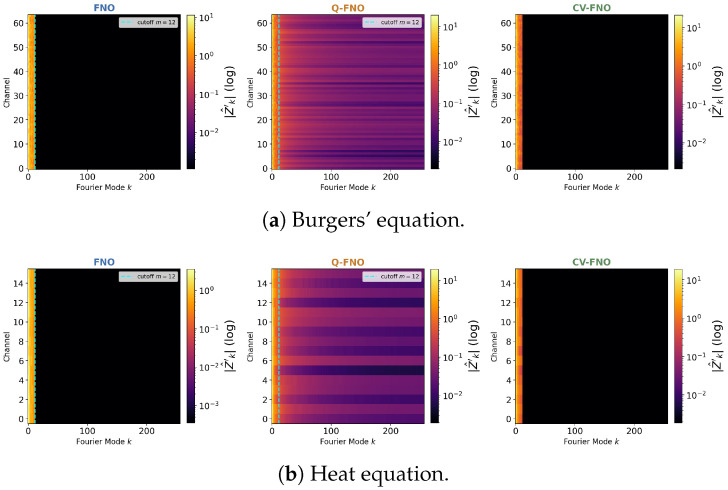
Spectral analysis: 1D benchmarks. Power spectra of the predicted solutions and ground truth for FNO, QFNO, and CV-QFNO. The classical FNO and the proposed CV-QFNO exhibited consistent spectral behaviour: both reproduced the relevant low-frequency content and enforced a sharp truncation beyond the retained Fourier modes. In contrast, the QFNO displayed nonzero spectral contributions beyond the cutoff, visible as horizontal channel-dependent bands, due to the non-suppressed Fourier modes inherited from the parallel qubit construction.

Figure 5 provides a direct spectral diagnostic of the learned operators. In both benchmarks, the CV-QFNO reproduced the reference spectrum with essentially the same fidelity as the FNO and confined the spectral activation to the retained modes.

As explained in Section 2.4, the experiments were performed as classical PyTorch 2.7.0 simulations of the corresponding CV operations. Therefore, the mode-isolation step was implemented numerically by setting the non-selected Fourier modes to zero, which is the classical linear-algebra equivalent of routing those modes through CV loss channels with transmissivity η=0. The resulting spectra show that this simulated loss-channel mechanism preserved the truncated spectral inductive bias of the classical FNO without introducing spurious spectral artefacts.

### 3.2. Two-Dimensional Experiments

We next considered two two-dimensional operator-learning benchmarks: the incompressible Navier–Stokes equation in vorticity form,

(48) $$\partial_{t}\omega \left(x,y,t\right)+\left(u(x,y,t)\cdot \nabla \right)\omega \left(x,y,t\right)=\nu \;\Delta \omega \left(x,y,t\right),\;\left(x,y\right)\in {\left[0,1\right]}^{2},\;t\in \left(0,T\right],$$

and the Darcy flow equation,

(49) $$-\nabla \cdot \;\left(a(x,y)\;\nabla u(x,y)\right)=f\left(x,y\right),\;\left(x,y\right)\in {\left[0,1\right]}^{2}.$$

Both problems are posed on the two-dimensional spatial domain $D={\left[0,1\right]}^{2}$, so d=2, but they represent different classes of PDE operators. In Equation (48), ω denotes the scalar vorticity, $u=(u_{x},u_{y})$ is the divergence-free velocity field, Δω is the spatial Laplacian of the vorticity, and ν>0 is the kinematic viscosity. The velocity is recovered from the vorticity through the Biot–Savart relation. In Equation (49), a(x,y) is the spatially varying permeability, u(x,y) is the pressure field, ∇u is the pressure gradient, and f(x,y) is a prescribed source term.

We used N=30 trajectories at $\nu =10^{-3}$ for Navier–Stokes, generated with a pseudo-spectral solver on a 256×256 grid over 40 time steps, and N=500 input–output pairs for Darcy flow, solved on a 421×421 finite-difference grid. The permeability was a thresholded Gaussian random field with spectral density $(4\pi^{2}{\left|k\right|}^{2}+\tau^{2}{)}^{-\alpha }$, α=2, τ=3, taking values a∈{3,12}.

For the Navier–Stokes benchmark, the learning task was formulated as a one-step time-evolution problem. The input consisted of the spatial coordinates concatenated with $T_{\mathrm{in}}$ consecutive vorticity snapshots,

(50) $$a\left(x,y\right)=\left(x,\;y,\;\omega \left(x,y,t_{1}\right),\ldots ,\omega \left(x,y,t_{T_{\mathrm{in}}}\right)\right)\in R^{d_{a}},\;d_{a}=T_{\mathrm{in}}+2,$$

and the target output was the vorticity at the next time step,

(51) $$u\left(x,y\right)=\omega \left(x,y,t_{T_{\mathrm{in}}+1}\right)\in R^{d_{u}},\;d_{u}=1.$$

Thus, the learned operator approximated the local-in-time evolution map

$$G^{†}:\left(x,\;y,\;\omega \left(\cdot ,t_{1}\right),\ldots ,\omega \left(\cdot ,t_{T_{\mathrm{in}}}\right)\right)\mapsto \omega \left(\cdot ,t_{T_{\mathrm{in}}+1}\right).$$

For the Darcy flow, the learning task was instead static. The input was the permeability field concatenated with the spatial coordinates,

(52) $$a\left(x,y\right)=\left(x,\;y,\;a(x,y)\right)\in R^{d_{a}},\;d_{a}=3,$$

and the target output was the scalar pressure solution,

(53) $$u\left(x,y\right)\in R^{d_{u}},\;d_{u}=1.$$

The corresponding solution operator was

$$G^{†}:a\left(\cdot ,\cdot \right)\mapsto u\left(\cdot ,\cdot \right),$$

where a(x,y) denotes the permeability coefficient in Equation (49). This elliptic operator is nonlocal: local changes in the permeability can affect the pressure solution across the whole domain.

In the implementation, both benchmarks were evaluated on a 64×64 uniform grid. After the lifting map, the latent field was represented by the tensor $A\in R^{N_{c}\times N_{x}\times N_{y}}$ with $N_{x}=N_{y}=64,$ as introduced in Equation (8), where $N_{c}$ was the latent channel dimension. The Fourier transform was applied along the two spatial directions (see Equation (25)) while the spectral mixing acted along the channel dimension for each retained Fourier mode pair $\left(k_{1},k_{2}\right)$. This is the two-dimensional spectral structure used by the classical FNO and reproduced, with constrained orthogonal mixing, by the QFNO and CV-QFNO architectures.

Both datasets were subsampled for training: factor 4 to 64×64 for Navier–Stokes ($N_{\mathrm{train}}=25$, $N_{\mathrm{test}}=5$, 100 epochs) and factor 5 to 85×85 for Darcy flow ($N_{\mathrm{train}}=400$, $N_{\mathrm{test}}=50$, 150 epochs).

The different dataset sizes reflect the different structure of the two learning problems. In the time-stepping formulation of Navier–Stokes, consecutive vorticity fields are strongly correlated, the dynamics are smooth over short time intervals, and each trajectory contributes multiple input–output pairs to the effective training set. Darcy flow, by contrast, is a static elliptic problem: each input is an independent realisation of a thresholded Gaussian random permeability field a(x,y)∈{3,12} paired with the corresponding pressure solution u(x,y). Since there is no temporal structure to exploit, and since the elliptic solution operator a↦u is nonlocal through the associated Green’s operator, a larger ensemble is required to represent the input distribution adequately.

**Figure 6:**
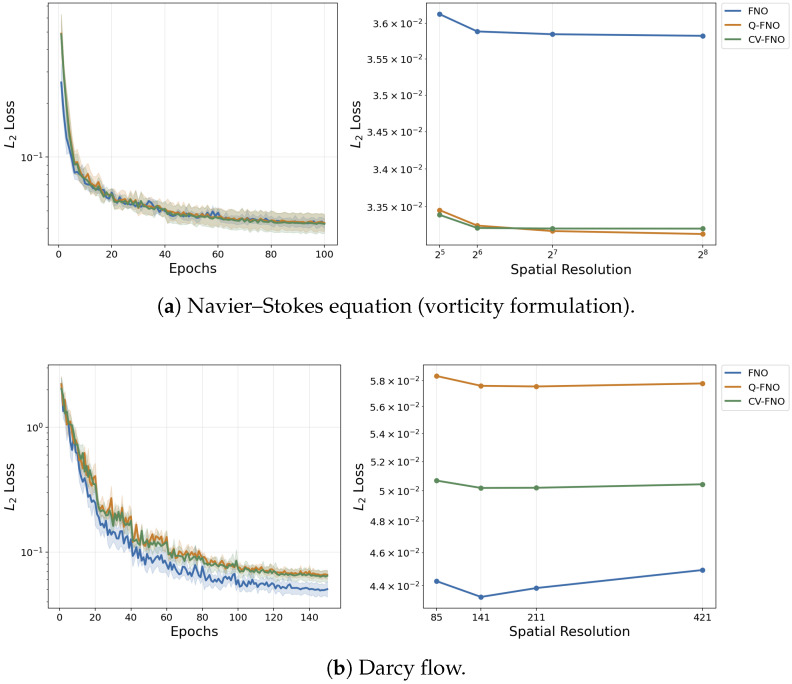
Training dynamics and resolution generalisation: 2D benchmarks. Left: test loss evolution over training epochs. Each solid line shows the geometric mean of the relative $\ell_{2}$ loss across four independent runs (seeds 0, 11, 22, 33); the shaded band spans ±1 geometric standard deviation across seeds. Right: relative $\ell_{2}$ test loss as a function of spatial resolution, evaluated zero-shot without retraining.

Both benchmarks were trained with the Adam optimiser, using an initial learning rate of $2\times 10^{-3}$, reduced by a factor of two every 20 epochs. As in the one-dimensional case, all the two-dimensional experiments were repeated across four independent random seeds (0, 11, 22, 33); the loss curves in Figure 6 show the geometric mean with ±1 geometric standard deviation band, and the converged test errors are reported in Table 4c,d. On Navier–Stokes, all three architectures converged to statistically equivalent accuracy: the FNO reached $\left(4.32\pm 0.23\right)\times 10^{-2}$, the QFNO $\left(4.37\pm 0.47\right)\times 10^{-2}$, and the CV-QFNO $\left(4.31\pm 0.51\right)\times 10^{-2}$, with differences well within the cross-seed variability. The quantum-inspired models achieved this with 4.1× fewer parameters (223,797 versus 926,517 for the FNO). On the Darcy flow, the FNO converged to $\left(5.10\pm 0.59\right)\times 10^{-2}$, while the QFNO and CV-QFNO reached $\left(6.73\pm 0.53\right)\times 10^{-2}$ and $\left(6.55\pm 0.68\right)\times 10^{-2}$ respectively, again with a 4.1× parameter reduction (910,977 versus 3,698,817). In this static elliptic setting, the orthogonal constraint did not match the accuracy of the unconstrained FNO; the difference between the CV-QFNO and the QFNO was small and lay within the cross-seed variability. Nevertheless, the orthogonal parameterisation produced a more stable optimisation trajectory: the average cross-seed log-standard deviation of the test loss, computed epoch-by-epoch over the full training run, was 0.154 for the FNO, compared with 0.109 for the QFNO and 0.128 for the CV-QFNO. This pattern mirrors the one observed for Burgers’ equation in the one-dimensional case, suggesting that the orthogonal constraint consistently reduces sensitivity to random initialisation, even when it does not yield a lower final test error.

### 3.3. Summary of Benchmark Results

Table 4 collects the converged test errors for all the models across the four benchmarks. For each model and benchmark, four independent runs were performed with different random seeds (0, 11, 22, 33); within each run the test loss was averaged over the last 20 epochs to obtain a stable plateau estimate, and the reported mean and standard deviation were computed across these four per-seed values.

Across all the experiments, the QFNO and CV-QFNO used 4.1× fewer trainable parameters than the FNO (1D Burgers’: 122,561 vs. 419,009; 1D heat: 9329 vs. 28,145; 2D Navier–Stokes: 223,797 vs. 926,517; 2D Darcy: 910,977 vs. 3,698,817), a direct consequence of restricting the spectral-mixing matrices to the orthogonal group.

For Burgers’ equation, all three models reach comparable accuracy, with the FNO attaining a marginally lower mean error. For the heat equation, the orthogonal constraint acted as an implicit regulariser: the FNO exhibited overfitting (its test loss exceeded its training loss on all four seeds), whereas the QFNO achieved the best test error of $\left(2.85\pm 0.17\right)\times 10^{-2}$ and the CV-QFNO remained close to the FNO at $\left(3.39\pm 0.11\right)\times 10^{-2}$. For Navier–Stokes, the three architectures converged to statistically equivalent errors, (4.32±0.23), (4.37±0.47), and $\left(4.31\pm 0.51\right)\times 10^{-2}$ for the FNO, QFNO, and CV-QFNO respectively, so the orthogonal parameterisation matched classical accuracy with a 4.1× parameter reduction. For the Darcy flow, the FNO retained a clear accuracy advantage ($\left(5.10\pm 0.59\right)\times 10^{-2}$) over the constrained models ((6.73±0.53) and $\left(6.55\pm 0.68\right)\times 10^{-2}$ for the QFNO and CV-QFNO); the difference between the two quantum-inspired models was small relative to the cross-seed variability.

Taken together, the results confirm that the CV-QFNO constitutes a viable photonic analogue of the FNO spectral layer: it matched or closely approached classical accuracy on three out of the four benchmarks, systematically using 4.1× fewer parameters, and it provided a hardware-compatible route to a photonic implementation of the FNO spectral mechanism.

This behaviour can be understood from the spectral properties of the benchmark problems. As discussed in Section 2.2 and as illustrated in Figure 5, the QFNO does not exactly implement the FNO spectral truncation: Fourier modes outside the retained band are not suppressed but are carried unchanged through the circuit, leading to residual high-frequency activations. In the present benchmarks, however, the solutions are relatively smooth so their Fourier energy decays rapidly with the mode index. As a result, most of the relevant dynamics are already contained in the retained modes, while the unsuppressed modes carry only a small amount of energy. Their effect on the final prediction is therefore limited, and the learnable orthogonal weights can partly compensate for their presence during training.

The η=0 loss-channel mechanism of the CV-QFNO removes this mismatch by enforcing the desired spectral truncation by construction, rather than relying on training to reduce the influence of the extra modes. Its advantage is therefore expected to become more visible in regimes where important dynamics extend to higher Fourier modes, such as low-viscosity Burgers’ equation, high-Reynolds-number Navier–Stokes flows, or deeper architectures where small spectral errors may accumulate across stacked Fourier blocks. The benchmarks considered here mostly lie in a smooth, spectrally compact regime, which explains why the difference between the CV-QFNO and QFNO remained moderate across all four benchmarks.

## 4. Discussion

The results presented in this work should be interpreted primarily as an architectural and algorithmic validation of the proposed CV-QFNO framework. Although the construction was motivated by CV quantum computing and by its natural photonic implementation, all the numerical experiments were performed as classical simulations. Every optical or quantum operation was replaced by its matrix-algebraic equivalent and executed on a GPU. The resulting pipeline is therefore a classical algorithm, and no quantum or photonic hardware is involved in the reported results. Consequently, the experiments should not be interpreted as a demonstration of quantum computational advantage.

This distinction is particularly important in the present Gaussian setting. Gaussian CV circuits admit efficient classical descriptions in terms of covariance matrices, and the simulations performed in this work exploited exactly this linear-algebraic structure. The value of the proposed model therefore does not lie in outperforming classical neural operators on conventional hardware but in showing that the spectral mechanism of the FNO admits a coherent CV photonic formulation. In particular, the CV-QFNO identifies a direct optical analogue of the three operations that define the FNO spectral layer: Fourier transformation, mode selection, and channel mixing. Within this formulation, the η=0 loss-channel mechanism provides a principled photonic counterpart of the mode-zeroing step of the classical FNO, resolving the spectral mismatch of the qubit-based QFNO.

The numerical results support this interpretation. Across the one- and two-dimensional benchmarks, the CV-QFNO achieved predictive accuracy comparable to the classical FNO and the qubit-based QFNO while using a substantially smaller number of trainable parameters. It should be emphasised, however, that the observed accuracy differences are entirely attributable to two classical algorithmic choices: the orthogonal constraint on the spectral-mixing matrices and the exact spectral truncation of non-retained Fourier modes. The role of the CV photonic formulation is not to introduce a new algorithmic mechanism but to provide a physically grounded motivation for these constraints, showing that they arise naturally from Gaussian photonic operations. Consequently, the parameter reduction should not be interpreted as a wall-clock speedup: in the present simulations, quantum and photonic operations were emulated through classical linear algebra, and training-time comparisons would mix the effect of a smaller parameter count with the overhead of constructing the orthogonal mixing matrices. The practical relevance of this blueprint lies in regimes that classical simulation cannot access: when the problem is natively continuous-variable or quantum in nature, the CV-QFNO could operate directly on physical quantum states, with entanglement between registers and non-Gaussian resources enabling spectral interactions outside the efficiently simulable Gaussian sector, potentially opening pathways toward quantum operator learning with no efficient classical counterpart.

The spectral diagnostics further clarify the structural difference between the architectures. The classical FNO and the proposed CV-QFNO both enforce a hard cutoff beyond the retained Fourier modes. By contrast, the parallel qubit-QFNO construction leaves non-selected modes unchanged, which produces residual spectral activations beyond the cutoff. This behaviour is visible in the power spectra and directly reflects the spectral mismatch analysed in Section 2.2. The CV-QFNO removes this mismatch by suppressing non-retained modes through zero-transmissivity loss channels before the inverse Fourier transform.

A second limitation concerns hardware realisability. Although the CV-QFNO removes the entangling-gate compilation overhead of the qubit formulation, a full photonic implementation of the architecture remains beyond current integrated photonic capabilities. The required number of optical modes, parallel branches, Gaussian transformations, loss channels, and readout stages would pose substantial experimental challenges. Moreover, training remains a hybrid process in which the optimisation of the trainable parameters is performed classically. Any advantage at the circuit level would therefore need to be assessed together with the cost of data preparation, readout, and classical optimisation. A full hardware analysis, including squeezing requirements, optical loss budgets, interferometer depth, and measurement noise, is deferred to future work targeting natively quantum or continuous-variable problems, where such a characterisation becomes most meaningful.

For the classical PDE benchmarks considered here, standard neural operators remain the most practical approach. They are accurate, computationally efficient, and directly deployable on conventional hardware. The parameter reduction observed in the CV-QFNO is structurally interesting and reflects the orthogonal parameterisation of the spectral layer, but it does not by itself provide a sufficient practical reason to replace classical FNOs with photonic hardware for these tasks. The main significance of the present work is therefore conceptual: it establishes a faithful CV photonic blueprint for the FNO spectral layer and clarifies how a quantum-photonic operator-learning architecture can reproduce the truncated Fourier inductive bias of the classical model.

## 5. Conclusions

This work introduces the Continuous-Variable Quantum Fourier Neural Operator (CV-QFNO), a Gaussian photonic analogue of the spectral layer of the classical Fourier Neural Operator. Building on bipartite Gaussian encoding and the optical quantum Fourier transform, the CV-QFNO represents latent fields in the covariance structure of Gaussian states and maps Fourier-domain processing, mode selection, and channel mixing onto native continuous-variable optical operations.

The main architectural contribution is a CV spectral mixing layer that recovers the truncated spectral structure of the classical FNO. In the one-dimensional setting, each retained Fourier mode is processed in an independent optical branch: non-selected modes are removed through loss channels with transmissivity η=0, while a pyramidal interferometer applies a learnable orthogonal transformation across the channel dimension. The construction is then extended to two spatial dimensions through a cross-circuit interferometric coupling, providing a photonic generalisation of the same mode-wise principle.

This formulation addresses two limitations of qubit-based QFNOs. First, it avoids the compilation overhead associated with RBS gates, since the corresponding transformations are native linear-optical operations in the CV setting. Second, it removes the spectral mismatch of the parallel qubit-QFNO architecture, where non-selected Fourier modes remain unchanged, by suppressing them directly through η=0 loss channels. As a result, the CV-QFNO restores the hard spectral truncation that defines the classical FNO layer.

We also introduce a matrix-exponential parameterisation of the orthogonal spectral-mixing matrices, which preserves the number of trainable parameters of the Givens construction while avoiding an explicit sequential product of rotations during classical training. Our numerical experiments on Burgers’, heat, Navier–Stokes, and Darcy-flow benchmarks showed that the CV-QFNO achieves competitive predictive accuracy with the FNO and QFNO, while using substantially fewer trainable parameters than the classical FNO. The spectral diagnostics confirmed the key structural feature of the CV construction: the CV-QFNO reproduces the hard cutoff of the classical FNO spectrum, whereas the QFNO retains residual activations beyond the selected modes.

Overall, the CV-QFNO provides a coherent Gaussian photonic formulation of Fourier-domain operator learning. Rather than demonstrating immediate computational advantage, the present work defines an architectural blueprint for photonic neural operators.

The most promising future directions concern settings in which the input data are themselves optical or quantum in nature, rather than classical fields already discretised on a digital grid. For natively optical inputs, such as coherent signals generated by interferometers, wavefront sensors, or free-space optical communication systems, a photonic implementation of the CV-QFNO could in principle operate directly on the incoming field, reducing or bypassing the analogue-to-digital conversion and classical encoding overheads. For intrinsically quantum inputs, as in quantum sensing, quantum simulation, or quantum communication, the bipartite CV architecture could act directly on state-level correlations rather than on classical samples extracted from them.

A longer-term route toward computational regimes beyond efficient classical emulation would require moving beyond the Gaussian framework. Non-Gaussian resources, such as cubic-phase operations or photon-number-resolving measurements, could introduce nonlinear spectral interactions outside the efficiently simulable Gaussian sector. Exploring these directions, together with higher-dimensional generalisations of the architecture and applications to more complex time-dependent problems, represents a natural next step for this line of research. 

## Figures and Tables

**Table 1:** Circuit resources per phase of a single Parallel QFNO layer. Each RBS gate compiles to 2 CZ gates (Equation (21)). Encoding counts from [15]; QFT/IQFT butterfly from [5].

Phase	Depth	RBS Gates	CZ Gates
Encoding	$\log_{2}N_{c}+2N_{c}\log_{2}N_{s}$	$\left(N_{c}-1\right)+\left(2N_{c}-1\right)\left(N_{s}-1\right)$	$2[\left(N_{c}-1\right)+\left(2N_{c}-1\right)\left(N_{s}-1\right)]$
Unary-QFT	$\log_{2}N_{s}$	$\frac{N_{s}}{2}\log_{2}N_{s}$	$N_{s}\log_{2}N_{s}$
QFL (*K* parallel circuits)	$2N_{c}-3$	$K\cdot \frac{N_{c}\left(N_{c}-1\right)}{2}$	$K\cdot N_{c}\left(N_{c}-1\right)$
Unary-IQFT	$\log_{2}N_{s}$	$\frac{N_{s}}{2}\log_{2}N_{s}$	$N_{s}\log_{2}N_{s}$
Total	$O(N_{c}\log N_{s})$	$O(KN_{c}^{2}+N_{c}N_{s}+N_{s}\log N_{s})$	

**Table 2:** Optical resources per phase of a single CV-QFNO layer (*K* parallel circuits). BS denotes a passive beam splitter; the encoding step additionally requires $r=\min(N_{c},N_{s})$ two-mode squeezers (depth 1). Interferometer counts use the Clements rectangular decomposition [21].

Phase	Depth	Beam Splitters
TMS-SVD encoding (*U*, Σ, *V*)	$1+\max(N_{c},\;N_{s})$	$\frac{N_{c}\left(N_{c}-1\right)}{2}+\frac{N_{s}\left(N_{s}-1\right)}{2}$
CV-QFT_*c*_	$\log_{2}N_{s}$	$\frac{N_{s}}{2}\log_{2}N_{s}$
Loss channels (*K* circuits)	1	$K(N_{s}-1)$
CV-QFL (*K* parallel pyramidal)	$2N_{c}-3$	$K\cdot \frac{N_{c}\left(N_{c}-1\right)}{2}$
CV-IQFT_*c*_	$\log_{2}N_{s}$	$\frac{N_{s}}{2}\log_{2}N_{s}$
Total	$O(\max\left(N_{c},N_{s}\right))$	$O(KN_{c}^{2}+N_{c}^{2}+N_{s}^{2})$

**Table 3:** Optical resources per phase of a single CV-QFNO layer in the 2D case ($K_{x}K_{y}$ retained mode pairs, $N_{c}$ channels). Depths of phases acting on disjoint registers are computed in parallel; the sequential circuit depth is dominated by the encoding and the channel mixing interferometer. Interferometer counts use the Clements rectangular decomposition [21].

Phase	Depth	Beam Splitters (Total)
TMS-SVD encoding ($N_{c}$ circuits per branch)	$1+\max(N_{x},\;N_{y})$	$N_{c}K_{x}K_{y}\;\left[\frac{N_{x}\left(N_{x}-1\right)}{2}+\frac{N_{y}\left(N_{y}-1\right)}{2}\right]$
2D CV-QFT (both registers in parallel)	$\max(\log_{2}N_{x},\;\log_{2}N_{y})$	$N_{c}K_{x}K_{y}\;\left[\frac{N_{x}}{2}\log_{2}N_{x}+\frac{N_{y}}{2}\log_{2}N_{y}\right]$
Loss channels ($r_{x}$ and $r_{y}$)	1	$N_{c}K_{x}K_{y}\;\left(N_{x}+N_{y}-2\right)$
Channel mixing $O(\theta_{k_{1},k_{2}})$ ($K_{x}K_{y}$ interferometers)	$2N_{c}-3$	$K_{x}K_{y}\cdot \frac{N_{c}\left(N_{c}-1\right)}{2}$
2D CV-IQFT (both registers in parallel)	$\max(\log_{2}N_{x},\;\log_{2}N_{y})$	$N_{c}K_{x}K_{y}\;\left[\frac{N_{x}}{2}\log_{2}N_{x}+\frac{N_{y}}{2}\log_{2}N_{y}\right]$
Total	$O(\max\left(N_{x},N_{y},N_{c}\right))$	$O\;\left(N_{c}K_{x}K_{y}\left(N_{x}^{2}+N_{y}^{2}\right)+K_{x}K_{y}N_{c}^{2}\right)$

**Table 4:** Convergence summary. Relative $\ell_{2}$ test error at convergence for all four benchmarks. Each entry was computed from four independent training runs with different random seeds: for each seed the test loss was averaged over the last 20 epochs to obtain a per-seed converged value, and the reported mean ± standard deviation was then taken across the four seeds.

Model	Relative $\ell_{2}$ (Mean ± Std)
(a) Burgers’ equation (1D)	
FNO	$\left(2.52\pm 0.19\right)\times 10^{-2}$
QFNO	$\left(2.97\pm 0.13\right)\times 10^{-2}$
CV-QFNO	$\left(2.93\pm 0.16\right)\times 10^{-2}$
(b) Heat equation (1D)	
FNO	$\left(3.27\pm 0.10\right)\times 10^{-2}$
QFNO	$\left(2.85\pm 0.17\right)\times 10^{-2}$
CV-QFNO	$\left(3.39\pm 0.11\right)\times 10^{-2}$
(c) Navier–Stokes (2D)	
FNO	$\left(4.32\pm 0.23\right)\times 10^{-2}$
QFNO	$\left(4.37\pm 0.47\right)\times 10^{-2}$
CV-QFNO	$\left(4.31\pm 0.51\right)\times 10^{-2}$
(d) Darcy flow (2D)	
FNO	$\left(5.10\pm 0.59\right)\times 10^{-2}$
QFNO	$\left(6.73\pm 0.53\right)\times 10^{-2}$
CV-QFNO	$\left(6.55\pm 0.68\right)\times 10^{-2}$

## Data Availability

The code and data supporting the findings of this study are available in the GitHub repository (https://github.com/PaoloMarcandelli, accessed on 25 June 2026) of the corresponding author. They are currently private and will be made publicly available upon publication of this article.
